# DEMENTIA RISK ATTRIBUTED TO THREE METABOLIC FACTORS: A BURDEN OF PROOF STUDY

**DOI:** 10.1093/geroni/igae098.2353

**Published:** 2024-12-31

**Authors:** Anh Thy Nguyen, Yvonne Yiru Xu, Xiaochen Dai, Christian Razo, Liane Ong, Natalie Chen, Channa Buxbaum, Jaimie Steinmetz

**Affiliations:** University of Washington, Seattle, Washington, United States; University of Washington, Seattle, Washington, United States; Institute for Health Metrics and Evaluation, Seattle, Washington, United States; University of Washington, Seattle, Washington, United States; Institute for Health Metrics and Evaluation, Seattle, Washington, United States; University of Washington, Seattle, Washington, United States; University of Washington, Seattle, Washington, United States; University of Washington, Seattle, Washington, United States

## Abstract

Hypertension, diabetes, and obesity are three widely recognized drivers of dementia risk. However, the dose-response relationships between continuous indicators of these conditions and dementia incidence and mortality have not been quantified systematically. The Burden of Proof methodology, a meta-analytic framework developed by the Institute for Health Metrics and Evaluation, enables a robust estimation of these relationships along exposure ranges, considers between-study heterogeneity, and enables comparisons of the strength of evidence between risk-outcome pairs. Using this framework, we estimated the mean relative risk (RR) and uncertainty intervals (UI) that account for between-study heterogeneity for systolic blood pressure (SBP; 51 studies), fasting plasma glucose (FPG; 11 studies), and body mass index (BMI; 15 studies). For SBP, compared to 100 mmHg, the estimated mean RR for dementia was 1.03 (95% UI=1.00-1.07) at 140 mmHg (threshold for hypertension), increasing to 1.17 (95% UI=1.01-1.37) at 180 mmHg. At an FPG value of 7 mmol/L (consistent with diabetes diagnosis), mean RR was 1.31 (95% UI=1.07-1.61, compared to 4 mmol/L), increasing to 1.51 (95% UI=1.10-2.06) at 8 mmol/L (85th percentile of exposure). For BMI, mean RR estimates were above 1.00, although the estimates were not statistically significant. Our findings highlight the significant impact of elevated SBP and FPG levels on increasing dementia risk, underscoring the importance of managing blood pressure and glucose levels in the population. The marginal association between BMI and dementia merits further investigation to understand to its influence within the broader spectrum of dementia risk factors.

